# Hemicircumferential rectal endoscopic submucosal dissection combining multitraction and scope-dependent traction strategies

**DOI:** 10.1055/a-2344-7374

**Published:** 2024-07-03

**Authors:** Jean Grimaldi, Louis-Jean Masgnaux, Timothée Wallenhorst, Elena De Cristofaro, Jérôme Rivory, Jérémie Jacques, Mathieu Pioche

**Affiliations:** 1 Gastroenterology, Edouard Herriot Hospital, Lyon, France; 2 36684 Gastroenterology and Endoscopy Unit, University Hospital Centre Rennes, Rennes, France; 3 60259 Gastroenterology, University of Rome Tor Vergata Faculty of Medicine and Surgery, Rome, Italy; 4 Gastroenterology and Endoscopy Unit, CHU Dupuytren Limoges, Limoges, France

The development of endoscopic submucosal dissection (ESD) has long been hampered by the difficulty and length of the procedures involved. In recent years, the development of traction strategies has greatly facilitated the practice of ESD 11 22 . As part of our research into traction, we developed an adaptive multipolar traction strategy using the ATRACT device 33 44 55 . We wanted to test combining this strategy with a new endoscopic lifting device, the FlexLifter (Olympus, Japan), which uses forceps to pull the lesion above the plane of the endoscope, thereby enlarging the submucosal plane to be dissected.

We report the case of a 72-year-old patient referred for resection of a hemicircumferential granular laterally spreading tumor of the rectum measuring 130 × 95 mm. We first positioned the ATRACT 2+2 device ( Fig. 1Fig. 1 ) at the four cardinal points of the lesion and began dissection without the need to fix the rubber band to the opposite wall, owing to the amount of traction generated by the hemicircumferential shape of the lesion ( Fig. 2Fig. 2 ). We then used the FlexLifter device, which we first attached to the ATRACT rubber band ( Fig. 3Fig. 3 ) and then to a precise point on the edge of the lesion itself ( Video 1Video 1 ). As the tensile strength of the device diminished as the procedure progressed, we finally hooked the rubber band to the opposite rectal wall, before completing the dissection. The procedure took 78 minutes (speed 124.3 mm ^3^ /min) and there were no complications. The lesion was an intramucosal adenocarcinoma (Vienna 4.4) and an R0 resection was achieved.

**Fig. 1:**
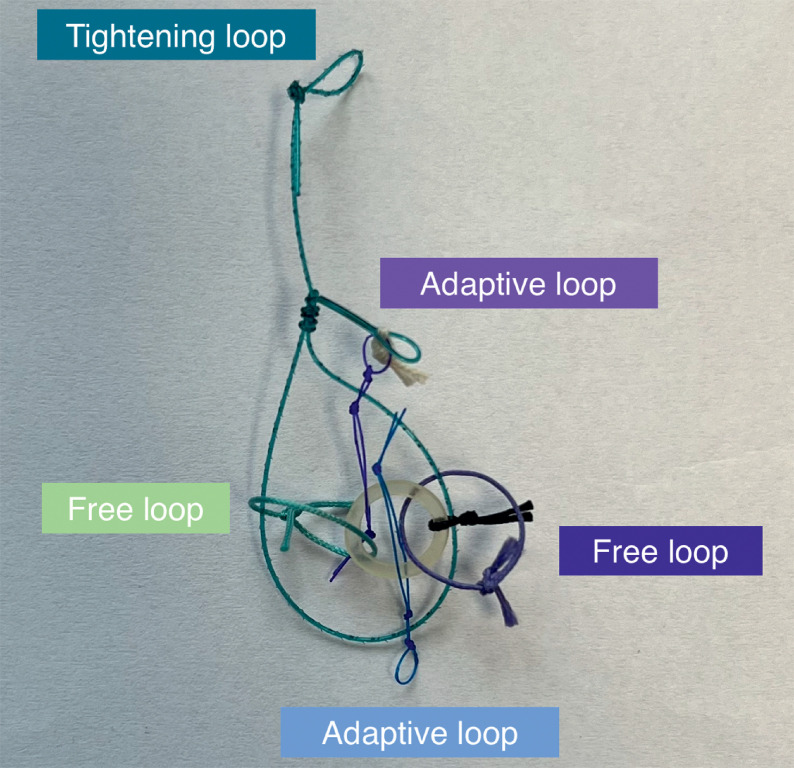
**Fig. 1** Photograph of the ATRACT 2+2 device.

**Fig. 2:**
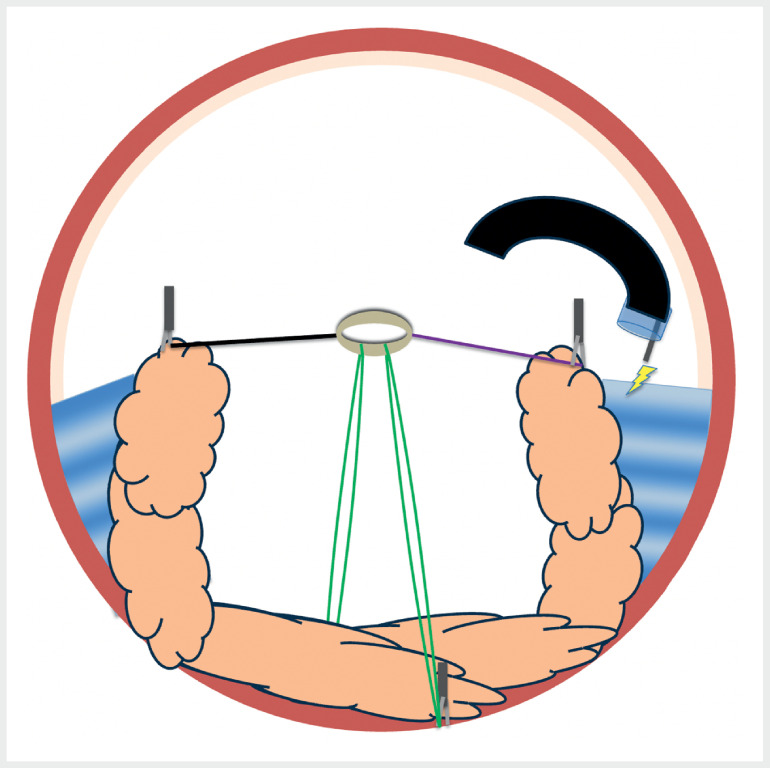
**Fig. 2** Schematic representation of an axial section of the rectum showing the multitraction device, which has been fixed at the four cardinal points, but that traction is sufficient to begin dissection without the need to fix the rubber band to the opposite wall, so this is kept for a later stage when traction decreases.

**Fig. 3:**
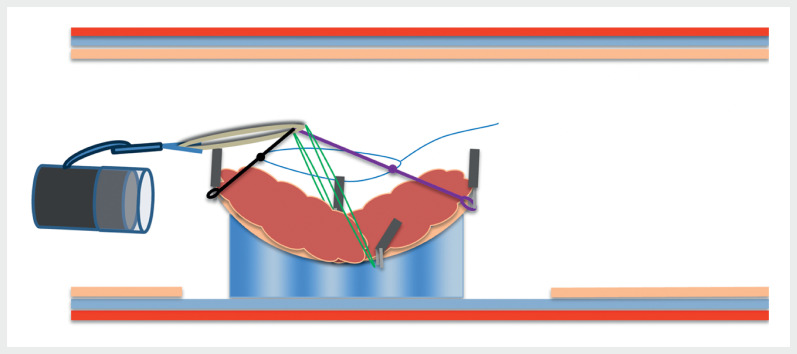
**Fig. 3** Schematic representation of scope-dependent traction using the FlexLifter device, which widens the submucosal plane by pulling the lesion margin above the plane of the scope.

A hemicircumferential rectal endoscopic submucosal dissection is performed with multipolar traction.Video 1Video 1

While the strategy of adaptive multipolar traction perpendicular to the lesion from the opposite wall seems the most effective, this case opens the door to the use of multimodal traction strategies adapted to each lesion.

Endoscopy_UCTN_Code_TTT_1AQ_2AD_3AD

## Notes

Endoscopy E-Videos https://eref.thieme.de/e-videos
*E-Videos* is an open access online section of the journal *Endoscopy* , reporting on interesting cases and new techniques in gastroenterological endoscopy. All papers include a high-quality video and are published with a Creative Commons CC-BY license. Endoscopy E-Videos qualify for HINARI discounts and waivers and eligibility is automatically checked during the submission process. We grant 100% waivers to articles whose corresponding authors are based in Group A countries and 50% waivers to those who are based in Group B countries as classified by Research4Life (see: https://www.research4life.org/access/eligibility/ ). This section has its own submission website at https://mc.manuscriptcentral.com/e-videos .

## References

[1] Bordillon P Pioche M Wallenhorst T Double-clip traction for colonic endoscopic submucosal dissection: a multicenter study of 599 consecutive cases (with video) Gastrointest Endosc 2021 94 333 343 33548280 10.1016/j.gie.2021.01.036

[2] Nagata M Advances in traction methods for endoscopic submucosal dissection: What is the best traction method and traction direction? World J Gastroenterol 2022 28 1 10.3748/wjg.v28.i1.1 35125817 PMC8793018

[3] Masgnaux LJ Grimaldi J Rivory J Endoscopic submucosal dissection assisted by adaptive traction: results of the first 54 procedures Endoscopy 2024 56 205 211 10.1055/a-2109-4350 37311544

[4] Grimaldi J Masgnaux LJ Rivory J Multipolar traction with adjustable force increases procedure speed during endoscopic submucosal dissection: the A-TRACT-4 traction device Endoscopy 2022 54 E1013 E1014 36002007 10.1055/a-1904-7666 PMC9736797

[5] Masgnaux LJ Grimaldi J Legros R Endoscopic submucosal dissection in the colon using a novel adjustable traction device: A-TRACT-2 Endoscopy 2022 54 E988 E989 10.1055/a-1888-3963 35926531 PMC9736814

